# Valuing tonsillitis manifestations in schoolchildren in Rio de Janeiro

**DOI:** 10.31744/einstein_journal/2022AO6342

**Published:** 2022-03-07

**Authors:** Rafaela Valentim Goldenzon, Tiago Oliveira Lucas, Maria de Marilacc Lima Roiseman, Marta Cristine Félix Rodrigues, Adriana Rodrigues Fonseca, Sheila Knupp Feitosa de Oliveira, Andréa Valentim Goldenzon

**Affiliations:** 1 Fundação Técnico-Educacional Souza Marques Escola de Medicina Souza Marques Rio de Janeiro RJ Brazil Escola de Medicina Souza Marques, Fundação Técnico-Educacional Souza Marques, Rio de Janeiro, RJ, Brazil.; 2 Universidade Federal do Rio de Janeiro Rio de Janeiro RJ Brazil Universidade Federal do Rio de Janeiro, Rio de Janeiro, RJ, Brazil.

**Keywords:** Tonsillitis, Pharyngitis, Rheumatic fever, Streptococcal infections, Rheumatic heart disease

## Abstract

**Objective:**

To evaluate the importance attributed to tonsillitis by guardians and their level of knowledge about the disease, correlating their management with their schooling and socioeconomic profiles.

**Methods:**

A quantitative, descriptive and observational cross-sectional study involving students aged 5 to 17 years from state-owned and private schools. A questionnaire was applied on management of tonsillitis and knowledge about rheumatic fever, addressing demographic and socioeconomic data.

**Results:**

A total of 323 students were included, predominantly females (61.3%), from state-owned schools (77.1%), with a mean age of 9.7±0.3 years. Among the guardians, 48.6% completed high school. Among the students 75.2% had at least one episode of odynophagia in the previous 12 months. Considering the previous 2 years, 89.8% reported this symptom. There was no collection of bacteriological specimens in 67.8%, and in 83% if taking into account only the last episode, despite the search for medical care in most cases (92.6%). The use of anti-inflammatory drugs was very frequent (43.0% “always” and 42.4% “sometimes”). Among the guardians, 81.7% denied being aware of the relation between rheumatic fever and inappropriate treatment of odynophagia; 85.8% said they wished they had received more information.

**Conclusion:**

Although it is quite common in schoolchildren, tonsillitis is neglected by many guardians and health professionals, and the ignorance of its relation with rheumatic fever is practically universal, which may contribute to its high prevalence.

## INTRODUCTION

Acute tonsillitis is a common disease caused by several viral and bacterial agents. Infection of the oropharynx caused by *Streptococcus pyogenes* requires specific identification and treatment due to the potential of triggering post-infectious complications.^(1)^ In Brazil, it is estimated that approximately 10 million cases of streptococcal tonsillitis occur every year, totaling 30,000 new cases of rheumatic fever, up to half of which can evolve with cardiac involvement.^(2)^ Despite this overwhelming estimate, no study was found specifically addressing the relevance attributed to tonsillitis or the population's knowledge about rheumatic fever and the management adopted by caregivers and physicians when facing tonsillitis.

The prevalence of rheumatic fever is the highest in individuals aged 5 to 15 years, and it is the leading cause of acquired heart disease in young adults in Brazil and many other developing countries. In 2015, the estimate of death from rheumatic heart disease was 319,400 people.^(3)^ The recent emergence of echocardiographic screening programs has shown a much higher prevalence of rheumatic heart disease in schoolchildren than previously described, suggesting estimates of incidence and prevalence of rheumatic fever in developing countries do not reflect the true impact of the disease.^(2)^ The major consequence of rheumatic heart disease for the health care system is reflected both in the frequent hospitalizations and required cardiac surgeries and in the functional impairment of these patients and their families.^(4)^ Recent guidelines from the World Health Organization (WHO) and the World Heart Federation (WHF) have committed to reduce by one-third the number of premature deaths from non-communicable diseases by 2030.^(5)^ Rheumatic heart disease is a severe complication of streptococcal tonsillitis. Rheumatic fever is a disease whose prevention is simple and low cost, and is carried out through the application of benzathine penicillin in patients with tonsillitis caused by group A beta-hemolytic *S. pyogenes*,^(6)^ a bacterium responsible for triggering the disease in genetically predisposed individuals and for approximately 20% to 30% of cases of tonsillitis in pediatric patients.^(7)^

The main reasons for inadequate treatment are lack of information about the origin of the disease, poor choice or irregular use of antibiotics, besides indiscriminate use of anti-inflammatory and other symptomatic drugs to manage oropharyngeal infection without identifying the causative agent. Thus, the resolution of clinical manifestations occurs, but with perpetuation of bacterial proliferation, which may subsequently cause the development of rheumatic fever.

Despite the estimated 10 million streptococcal tonsillitis cases per year in Brazil,^(2)^ no studies were found addressing its importance in the population or knowledge about its relation with rheumatic fever.

## OBJECTIVE

To evaluate the importance attributed to oropharyngeal infection by the guardians and the level of knowledge about the disease, correlating the management with the school and socioeconomic profiles.

## METHODS

This is a quantitative and descriptive cross-sectional study. The study was conducted based on an analysis of questionnaires completed during August 2018 by guardians of students aged 5 to 17 years, from two schools selected for their proximity to the educational institution of origin of the study. A total of 500 students in the chosen age group (120 from a private school and 380 from a state-owned school) received the set of documents consisting of the questionnaire (Table 1), a manual for filling it out, and an Informed Consent Form. The quantity of documents distributed was based on the proportion found between private and state-owned school students in the municipality of Rio de Janeiro, according to the *Instituto Nacional de Estudos e Pesquisas Educacionais Anísio Teixeira* (Inep).^(8)^ A total of 323 students were included, whose material was returned duly filled out within two weeks of receipt. Of those, 177 were excluded, 46 (38.3%) from the private school, and 131 (34.4%) from the state-owned school, for lack of response to the questionnaire.

**Table 1:** Questionnaire sent to the students’ guardians

Student:					
Male ( )			Date of birth: / /		
Female ( )					
Guardian:					
Guardian's level of schooling					
	( ) No schooling		( ) Secondary education (high school)		
	( ) 1^st^ to 4^th^ grade primary education		( ) Further education		
	( ) 5^th^ to 9^th^ grade primary education		( ) Graduate studies		
School					
	( ) School 1		( ) School 2		
	( ) School 3		( ) School 4		
Family monthly income					
	( ) Between 0 and R$ 1.254,00		( ) Between R$ 8.641,00 and R$ 11.261,00		
	( ) Between R$ 1.255,00 and R$ 2.004,00		( ) More than R$ 11.262,00		
	( ) Between R$ 2.005,00 and R$ 8.640,00				
How many times has your child had a sore throat over the last 12 months?					
	( ) None	( ) Once	( ) Twice	( ) Three times or more	
How many times has your child had a sore throat in prior years?					
	( ) None	( ) Once	( ) Twice	( ) Three times or more	
When your child has a sore throat, do you take him/her to the doctor?					
	( ) Always	( ) Never	( ) Sometimes		
When you take your child to the doctor, is specimen collected for a throat test?					
	( ) Always	( ) Never	( ) Sometimes		
When your child has a sore throat, is it treated with an anti-inflammatory?					
	( ) Always	( ) Never	( ) Sometimes		
When your child has a sore throat, is it treated with topical agents?					
	( ) Always	( ) Never	( ) Sometimes		
When your child has a sore throat, is it treated with an antibiotic?					
	( ) Always	( ) Never	( ) Sometimes		
The last time your child had a sore throat, did you take him/her to the doctor?				Yes	No
The last time your child had a sore throat, was specimen collected for a throat test?				Yes	No
The last time your child had a sore throat, was it treated with an anti-inflammatory?				Yes	No
The last time your child had a sore throat, was it treated with topical agents?				Yes	No
The last time your child had a sore throat, was it treated with an antibiotic?				Yes	No
The last time your child had a sore throat, did he/she have a fever?				Yes	No
The last time your child had a sore throat, did he/she have a cough?				Yes	No
The last time your child had a sore throat, did he/she have white patches in the throat?				Yes	No
The last time your child had a sore throat, did he or she have nodes (lumps, swelling) in the neck?				Yes	No
Have you ever heard of rheumatic fever?				Yes	No
Do you know someone who has been diagnosed with rheumatic fever?				Yes	No
Did you know that rheumatic fever is related to the inappropriate treatment of sore throats?				Yes	No
Would you like to receive information about rheumatic fever?				Yes	No

The research project was submitted to and approved by the Ethics Committee of the *Escola de Medicina Souza Marques da Fundação Técnico*-*Educacional Souza Marques*, under protocol # 2.834.886, and CAAE: 90753018.6.0000.5239.

For statistical analysis, the test of equality of two proportions was used to characterize the distribution of the relative frequency of each of the study variables. For the descriptive analysis of age, a 95% confidence interval was used.

The following data were collected: age, sex, school network (private or state-owned), schooling level of the guardian, family monthly income, and frequency of sore throat in the child over the past 12 months and in prior years, visits to the doctor for sore throat, associated symptoms, and their management of the symptom. In addition, the parents’ knowledge about rheumatic fever and the desire to receive information about the disease were questioned.

## RESULTS

The guardians of 323 students returned the questionnaires duly completed within the stipulated deadline (Table 2). The mean age of the students was 9.7±0.3 years, with a predominance of females (61.3%) and individuals from state-owned schools (77.1%). Regarding the guardians’ schooling level, the majority was composed of individuals who completed secondary education (high school) (48.6%). The families with income between zero and R$ 1.254,00 accounted for 32.2% of the sample (p=0.214). A minority of families had an income higher than R$ 8.641,00 (2.6%).

**Table 2:** Relative frequency of demographic variables

Variables		n=323 n (%)	p value
Sex			
	Female	198 (61.30)	<0.001
	Male	125 (38.70)	
School Network			
	Private	74 (22.90)	<0.001
	State-owned	249 (77.10)
Guardian's level of schooling			
	No schooling	5 (1.50)	<0.001
	1^st^ to 4^th^ grade	40 (12.40)	<0.001
	5^th^ to 9^th^ grade	78 (24.10)	<0.001
	Secondary education (high school)	157 (48.60)	Reference
	Graduate studies	12 (3.70)	<0.001
	Further education	31 (9.60)	<0.001
Family monthly income		
	R$ 0.00 - R$ 1.254,00	104 (32.20)	0.214
	R$ 1.255,00 - R$ 2.004,00	47 (14.60)	<0.001
	R$ 2.005,00 - R$ 8.640,00	45 (13.90)	<0.001
	R$ 8.641,00 - R$ 11.261,00	5 (1.50)	<0.001
	>R$ 11.262,00	3 (0.90)	<0.001
	Unwilling to inform	119 (36.80)	Reference
How many times has your child had a sore throat over the last 12 months?			
	None	80 (24.80)	0.079
	Once	100 (31.00)	Reference
	Twice	87 (26.90)	0.259
	Three or more times	56 (17.30)	<0.001
How many times has your child had a sore throat in prior years?			
	None	33 (10.20)	<0.001
	One	72 (22.30)	<0.001
	Two	107 (33.10)	0.739
	Three or more	111 (34.40)	Reference

Of the total respondents, only 24.8% stated their child had not had any episode of sore throat in the past 12 months (p=0.079). For 31.0% of respondents, their child had a sore throat in the past 12 months once, and only 17.3% reported three or more episodes (p<0.001). Among the students assessed, 75.2% had had at least one episode of odynophagia in the previous 12 months. If the previous 2 years were considered, 89.8% reported this symptom. When analyzing the number of times the child had sore throat in the previous years, the most recurrent answers were three or more episodes (34.4%) and two episodes (33.10%).

As shown in table 3, the question “When you take your child to the doctor, is specimen collected for a throat test?” was the only question that had the highest percentage of response “never”, with 67.8% among the Likert scale type questions. The questions “When your child has a sore throat, is it treated with topical agents?” and “When your child has a sore throat, is it treated with an antibiotic?” had as most prevalent answer “sometimes”, with rates of 37.8% and 45.5%, respectively. As for the questions “When your child has a sore throat, do you take him/her to the doctor?” and “When your child has a sore throat, is it treated with an anti-inflammatory?” they had the answer “always” as the most recurrent, with 47.1% and 43.0%, respectively.

**Tabela 3:** Relative frequency of Likert-type

Variables		n (%)	p value
When your child has a sore throat, do you take him/her to the doctor?			
	Never	24 (7.4)	<0.001
	Sometimes	147 (45.5)	0.693
	Always	152 (47.1)	Reference
When you take your child to the doctor, is specimen collected for a throat test?			
	Never	220 (67.8)	Reference
	Sometimes	56 (17.3)	<0.001
	Always	48 (14.9)	<0.001
When your child has a sore throat, is it treated with an anti-inflammatory?			
	Never	47 (14.6)	<0.001
	Sometimes	137 (42.4)	0.874
	Always	139 (43.0)	Reference
When your child has a sore throat, is it treated with topical agents?			
	Never	106 (32.8)	0.188
	Sometimes	122 (37.8)	Reference
	Always	95 (29.4)	0.025
When your child has a sore throat, is it treated with an antibiotic?			
	Never	43 (13.3)	<0.001
	Sometimes	147 (45.5)	Reference
	Always	133 (41.2)	0.266

In the question “The last time your child had a sore throat, was specimen collected for a throat test?”, 83% answered “no.” In contrast, regarding the question “The last time your child had a sore throat, was it treated with an antibiotic?” 63.2% indicated “yes” (Table 4).

**Table 4:** Relative frequency of Yes/No

Question	Yes n (%)	No n (%)	p value*
The last time your child had a sore throat, did you take him/her to the doctor?	223 (69.0)	100 (31.0)	<0.001
The last time your child had a sore throat, was specimen collected for a throat test?	55 (17.0)	268 (83.0)	<0.001
The last time your child had a sore throat, was it treated with an anti-inflammatory?	204 (63.2)	119 (36.8)	<0.001
The last time your child had a sore throat, was it treated with topical agents?	157 (48.6)	166 (51.4)	0.479
The last time your child had a sore throat, was it treated with an antibiotic?	204 (63.2)	119 (36.8)	<0.001
The last time your child had a sore throat, did he/she have a fever?	210 (65.0)	113 (35.0)	<0.001
The last time your child had a sore throat, did he/she have a cough?	202 (62.5)	121 (37.5)	<0.001
The last time your child had a sore throat, did he/she have white patches in the throat?	77 (23.8)	246 (76.2)	<0.001
The last time your child had a sore throat, did he or she have nodes (lumps, swelling) in the neck?	62 (19.2)	261 (80.8)	<0.001
Have you ever heard of rheumatic fever?	157 (48.6)	166 (51.4)	0.479
Do you know someone who has been diagnosed with rheumatic fever?	60 (18.6)	263 (81.4)	<0.001
Did you know that rheumatic fever is related to the inappropriate treatment of sore throats?	59 (18.3)	264 (81.7)	<0.001
Would you like to receive information about rheumatic fever?	277 (85.8)	46 (14.2)	<0.001

* Test for equality of two proportions.

The majority had fever and cough associated with the condition (65.0% and 62.5%, respectively), and the minority had white patches in the throat and adenomegaly in the neck (23.8% and 19.2%, respectively).

When assessing the question “Did you know that rheumatic fever is related to the inappropriate treatment of sore throat?”, 81.7% denied having such knowledge. The question “Would you like to receive information about rheumatic fever?” was answered affirmatively by 85.8%.

## DISCUSSION

Although the incidence of rheumatic fever is more significant between 5 and 15 years of age, the study included patients from a wider age range, with students aged up to 17 years. Moreover, although the incidence is lower than in the mentioned age group, it is not null between 15 and 17 years, and has epidemiological significance.

According to the 2018 School Census released by Inep, participation of state-owned schools in the municipality of Rio de Janeiro (RJ) accounts to 67.08%,^(8)^ which is lower than in this study, in which 77.1% of students came from state-owned schools. In this group, there was a prevalence of guardian's schooling level up to high school, and low or not informed family monthly income. Thus, the restricted access to information reported in the study may reflect the socioeconomic panorama experienced by the families analyzed.

Sore throat is the second most common acute infectious manifestation observed by family physicians, and it is estimated that less than one in ten people with sore throat seek care.^(9)^ The high incidence of tonsillitis found in this study reflects such a panorama. When asked if they take their children to the doctor in case of sore throat, most said yes, showing a higher-than-expected valuation of the episodes. In Brazil, rapid tests are rarely available in the public health services,^(10)^ justifying the low frequency of its performance found in this study and the diagnosis based exclusively on clinical criteria. Thus, it is possible to conclude that most patients are treated empirically or, possibly, only according to these criteria.

Among the treatments used, anti-inflammatory drugs and antibiotics stood out. Although 70% to 85% cases of acute pharyngotonsillitis are viral,^(11)^ which would not justify the use of antibiotics, its almost universal use seems to reflect the low specificity or lack of knowledge of currently used diagnostic criteria. The indiscriminate use of antibiotics may be advantageous, initially to reduce the incidence of rheumatic fever, but it probably implies in the selection of resistant strains.

In the Centor score^(12)^ used to guide the need for testing and treatment for group A streptococcal pharyngitis in patients with oropharyngeal pain, adenomegaly, and tonsillar exudate are parameters that add one point to the score. Presence of cough, on the other hand, reduces one point, since it is a characteristic manifestation of viral infections. The fact that 62.5% reported coughing in the last episode suggests a higher incidence of viral infections. Despite this finding, most are treated with antibiotics.

Approximately half of the guardians had already heard of rheumatic fever, and many knew someone diagnosed with the disease. We observed that, despite the lack of knowledge on the part of the general population about the determinant factors for rheumatic fever, the diagnosis is sufficiently frequent so that is recognized, reflecting significant prevalence, functional repercussion, morbidity, and mortality in the young population.^(13)^

Few had prior knowledge of the relation between inappropriate treatment of streptococcic tonsillitis and rheumatic fever. The population's knowledge of rheumatic fever is very limited, and educational programs geared towards this topic may be beneficial in reducing the impact of rheumatic heart disease.^(14)^ Most of the guardians demonstrated interest in receiving information about rheumatic fever. In this way, it is possible to infer that the main factor for disinformation in the case of the disease under study is lack of dissemination of information to the population.

## CONCLUSION

The results obtained by evaluating the complete questionnaires confirmed the high incidence of tonsillitis in our midst, and the lack of knowledge regarding its relation with rheumatic fever. It is vital that the guardians value the complaint of odynophagia in their children, always seeking the orientation of healthcare professionals, who should be able to make the correct diagnosis and guarantee appropriate treatment. This disagrees with the picture found in this study, with frequent use of anti-inflammatory agents.

Most guardians demonstrated no knowledge of the relation between tonsillitis and rheumatic fever. The development of public measures to broaden knowledge of the population and healthcare professionals about the disease is also crucial, for there to be a greater valuation of the acute infection, allowing greater epidemiological control and reduced incidence of complications.
